# Asymptomatic Human Infections With Avian Influenza A(H5N1) Virus Confirmed by Molecular and Serologic Testing: A Scoping Review

**DOI:** 10.1001/jamanetworkopen.2025.40249

**Published:** 2025-10-29

**Authors:** Fatimah S. Dawood, Shikha Garg, Pragna Patel, Timothy M. Uyeki

**Affiliations:** 1 2024 Influenza A(H5N1) Response, Centers for Disease Control and Prevention, Atlanta, Georgia

## Abstract

**Importance:**

Since 1997, more than 1000 infections with highly pathogenic avian influenza A(H5N1) virus among humans have been reported globally. Given ongoing A(H5N1) outbreaks in animals, understanding the frequency of A(H5N1) virus infections among asymptomatic persons can inform public health risk assessments and infection prevention guidance.

**Objective:**

To identify and characterize reported cases of asymptomatic A(H5N1) virus infection among humans with confirmation by both molecular testing of 1 or more respiratory specimens and 1 or more serum specimens meeting World Health Organization criteria (molecularly and serologically confirmed [MSC]) or molecular confirmation (MC) alone.

**Evidence Review:**

MEDLINE, Embase, Global Health, Cochrane, Scopus, Virtual Health Library, and Europe PubMed Central were searched for publication through August 25, 2025. Articles for full-text screening were evaluated by 2 investigators. Studies published through August 25, 2025, were included if they reported a confirmed A(H5N1) virus infection that met MSC or MC criteria and had a full-text report in English. Articles were excluded if they reported results from A(H5N1) serologic testing alone, serosurveys, or other immunologic studies.

**Findings:**

Of 1567 unique reports that underwent title or abstract screening, 42 were selected for full-text screening, of which 10 met inclusion criteria (3 reports about 2 MSC cases and 7 reports about 16 MC cases). The 2 MSC cases occurred among adults in Pakistan and Vietnam who were identified by investigations of household contacts of index A(H5N1) case patients; 1 case patient also had exposure to A(H5N1) virus–infected chickens as the possible infection source and 1 is thought to have been infected through human-to-human transmission. Neither MSC case patient used personal protective equipment. Of 16 reported MC cases (14 adults, 2 children), 11 were identified by enhanced surveillance of persons exposed to A(H5N1) virus–infected poultry (8 in Bangladesh, 2 in Spain, and 1 in the UK) and the remaining 5 MC cases (3 in Vietnam, 2 in Cambodia) were identified by investigations of household contacts of index A(H5N1) case patients.

**Conclusions and Relevance:**

Asymptomatic human infections with A(H5N1) virus have been infrequently reported, with most cases identified through enhanced surveillance or household contact investigations of persons with known exposure. Robust data collection is needed from persons with possible asymptomatic A(H5N1) virus infection to inform future public health responses.

## Key Points

**Question:**

Have asymptomatic infections with highly pathogenic avian influenza A(H5N1) virus been reported in humans?

**Findings:**

This scoping review of published reports through August 25, 2025, identified 10 reports of 18 cases of asymptomatic infection with A(H5N1) virus, including 2 cases with molecular and serologic confirmation and 16 cases with molecular confirmation alone. Symptom ascertainment methods varied among reported cases.

**Meaning:**

Asymptomatic human avian influenza A(H5N1) virus infections have been infrequently reported, with most lacking serologic confirmation; prospective surveillance studies with serial respiratory and serum sampling and detailed symptom monitoring for persons with high-risk exposures could provide data to inform future public health responses.

## Teaser

This scoping review uses published reports through January 31, 2025, to identify and characterize reported cases of asymptomatic avian influenza A(H5N1) virus infection among humans with confirmation by both molecular testing of 1 or more respiratory specimen and 1 or more serum specimen.

## Introduction

Since 1997, more than 1000 human infections with highly pathogenic avian influenza A(H5N1) virus have been reported from 25 countries, largely among persons with unprotected exposures to infected animals.^1,2,3,4,5,6^ Given ongoing A(H5N1) outbreaks among animals, understanding the frequency of A(H5N1) virus infections among asymptomatic persons can inform public health risk and severity assessments^7^ and infection prevention guidance. Although cases of asymptomatic seasonal influenza virus infections among humans have been increasingly recognized,^8^ less is known about asymptomatic human infections with A(H5N1) virus. Efforts to identify asymptomatic A(H5N1) virus infections are hampered by interpretation of detection by either molecular or serologic testing alone^8^ because asymptomatic infection may not always induce detectable humoral immune responses and serosurveys often lack robust methods for symptom ascertainment. Detection by combined molecular and serologic testing among persons without symptoms provides more compelling evidence of true asymptomatic infection but may be less feasible in some settings, may miss cases, and is still vulnerable to gaps in symptom ascertainment.

## Methods

We conducted a scoping review of literature published on human cases of A(H5N1) using systematic search methods. Results are reported per the Preferred Reporting Items for Systematic Reviews and Meta-Analyses (PRISMA) reporting guideline,^9^ specifically for scoping reviews; the review protocol is available on request. The objective was to identify and characterize reported cases of asymptomatic A(H5N1) virus infection among humans with confirmation by both molecular testing of 1 or more respiratory specimens and 1 or more serum specimens meeting World Health Organization criteria^10^ (molecularly and serologically confirmed [MSC]) or molecular confirmation (MC) alone. The population of interest was children and adults worldwide. The primary outcome was infection with A(H5N1) virus without reported symptoms meeting MSC criteria. A secondary outcome was asymptomatic A(H5N1) virus infection with MC alone. This project was conducted under a nonresearch determination by the Centers for Disease Control and Prevention.

### Search Strategy

A medical librarian was consulted to develop a search strategy (eTable 1 and eTable 2 in Supplement 1). Results were included from searching MEDLINE, Embase, Global Health, Cochrane, Scopus, Virtual Health Library, and Europe PubMed Central with an initial search conducted on January 31, 2025, and a search update conducted on August 25, 2025. Results were deduplicated in EndNote, version 21 (Clarivate), and then manually during title screening. Articles selected for full-text screening were evaluated by 2 investigators (F.S.D. and T.M.U.) for inclusion, and reviewer conflicts were resolved through discussion.

### Inclusion Criteria

We included any study published through August 25, 2025, that reported a confirmed A(H5N1) virus infection that met primary or secondary outcome definitions and had a full-text report available in English. We excluded articles that reported results from A(H5N1) serologic testing in humans without molecular testing. We also excluded serosurveys and other immunologic studies,^11^ some of which have been interpreted as evidence of asymptomatic infection.

### Data Collection and Analysis

Data from eligible studies were extracted by 1 reviewer (F.S.D.) using an Excel, version 2024 (Microsoft Corp) template. Variables of interest for data abstraction included authors, publication year, journal, impetus for A(H5N1) virus testing, number of cases, case country, age (adult or child and age in years), suspected A(H5N1) virus exposures, specimen type for molecular testing (nasopharyngeal, oropharyngeal, or nasal swabs), reverse transcription–polymerase chain reaction (RT-PCR) cycle threshold value for A(H5N1)–positive specimens, duration of RT-PCR test positivity for A(H5N1) if serial specimens were collected, whether serologic testing was done and results, and antiviral treatment. Two reviewers (F.S.D. and T.M.U.) independently reviewed data abstraction and synthesis results. Results are described with meta-aggregative qualitative data synthesis as outlined in the JBI Manual for Evidence^12^ and are stratified by MSC or MC status.

## Results

### Identification of Publications

The initial search identified 1567 unique reports that underwent title or abstract screening after removal of duplicates. Forty-two reports met eligibility for full-text screening, of which 10 reports^13,14,15,16,17,18,19,20,21,22^ met full inclusion criteria and had data extracted. The 10 reports included 3 reports about 2 MSC cases and 7 reports about 16 MC cases (Figure). No additional reports were identified by reference list searches.

**Figure.**
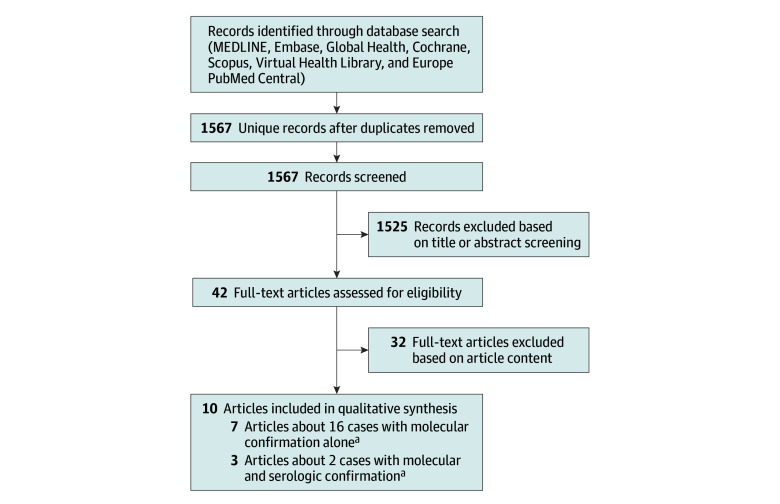
Flow Diagram of Screened and Included Reports ^a^Two articles described the same case.

### MSC Cases

The 2 MSC cases were reported in adults in Pakistan in 2007 and Vietnam in 2011; both cases were identified by detailed investigations of household contacts of index A(H5N1) case patients.^18,19^ The MSC asymptomatic case patient in Pakistan did not have exposure to sick or dead poultry and is thought to have acquired A(H5N1) virus infection through human-to-human transmission from exposure to 4 symptomatic siblings with A(H5N1) (3 confirmed cases and 1 probable case without A[H5N1] testing).^18^

The MSC asymptomatic case patient in Vietnam was a household member of a symptomatic confirmed A(H5N1) index case patient; both the index case patient and the asymptomatic case patient handled and slaughtered A(H5N1) virus–infected chickens before their infections were confirmed.^19^ The asymptomatic case patient had A(H5N1) virus isolated from a throat swab specimen collected 6 days after slaughtering chickens and then received oseltamivir postexposure prophylaxis (single dose 75 mg per day for 1 week), which was started for all household contacts of the index case patient after a contact investigation was started on the index case patient’s fifth illness day.

Neither of the 2 MSC case patients used personal protective equipment when in close contact with the index A(H5N1) case patients, or in the case of the case patient in Vietnam, with infected chickens.^18,19^ Both MSC case patients were confirmed seropositive for A(H5N1) virus antibodies based on convalescent serum specimens tested by microneutralization assay plus an additional serologic assay (Western blot for the case patient in Pakistan and hemagglutination inhibition assay for the case patient in Vietnam) (Table).^13,14,15,16,17,18,19,20,21,22^

**Table.** Reported Asymptomatic Cases of Highly Pathogenic Avian Influenza A(H5N1) Virus Detection by Molecular Testing of Upper Respiratory Tract Specimens With or Without Serologic Testing (N = 18)

Source	Country	Year of case detection	Reason for testing	Adult or child age, y^a^	Suspected source of A(H5N1) virus exposure	Respiratory specimen sources	A(H5) cycle threshold value	Serology	Antiviral receipt	No. of cases
**Molecular confirmation only**										
Olsen et al,^21^ 2005^b^	Vietnam	2004 (1), 2005 (2)	Epidemiologic contact investigation	Adults (36; 80; 61)	Household contact of case	NR	NR	NR	NR	3
Hassan et al,^17^ 2023	Bangladesh	2012-2013	Study of individuals working at live bird markets	Adult	Individual working at live bird market	NP and OP	NR	No	No	8
Oliver et al,^20^ 2022^c^	UK	2021-2022	National Avian Influenza surveillance program	Adult (80s)	Kept flock of Muscovy ducks inside and outside home	Nasal, OP	Mid-30s^d^	No	Yes (oseltamivir)^e^	1
Aznar et al,^14^ 2023	Spain	2022	National Avian Influenza surveillance program	Adults	Poultry workers on affected farm; egg collection and routine cleaning; removing dead hens, cleaning and disinfecting after outbreak of A(H5N1)	NP	35.8 and 34.6	Yes^f^	NR	2
Alexakis et al,^13^ 2024	Cambodia	2024 (July)	Epidemiologic contact investigation	Child	Dead chickens in backyard; household contact of case	NP and OP	NR	NR	Yes (oseltamivir)^e^	1
Fusaro et al,^16^ 2024	Cambodia	2024 (February)	Epidemiologic contact investigation	Child (16)	Household contact of case	NR	NR	NR	NR	1
**Molecular and serologic confirmation**										
*Weekly Epidemiologic Record*,^18^ 2008^g^	Pakistan	2007	Epidemiologic contact investigation	Adult (33)	Household contact of case (possible second-generation human-to-human transmission); no personal protective equipment used when caring for symptomatic family member with confirmed A(H5N1) virus infection	OP	NR	Yes^h^	No	1
Le et al,^19^ 2013	Vietnam	2011	Epidemiologic contact investigation	Adult (47)	Household contact of case; lived on commune with infected chickens; slaughtered chickens; no personal protective equipment used when in close contact with symptomatic family member with confirmed A(H5N1) virus infection or when handling and slaughtering infected chickens	OP	NR^i^	Yes^h^	Yes (oseltamivir)^e^	1

Abbreviations: NP, nasopharyngeal; NR, not reported; OP, oropharyngeal.

^a^ Age is presented in parentheses when reported.

^b^ Details on method of A(H5N1) case confirmation provided by personal communication with manuscript coauthor.

^c^ Case also reported by Capelastegui et al.^15^

^d^ Serial samples were positive on day 0, day 2, and day 7, where day 0 was the day of collection of the first positive respiratory sample.

^e^ The case patient in the UK received 1 day of oseltamivir prophylaxis with once-daily dosing on the day that the first nasal swab specimen was collected, followed by oseltamivir treatment with twice-daily dosing once the nasal swab specimen tested positive for A(H5N1) virus. The case patients in Cambodia received oseltamivir treatment. The case patient in Vietnam received oseltamivir prophylaxis with once-daily dosing.

^f^ Reported as negative by serologic testing but details of serologic assay and criteria for positive result were not reported.

^g^ Case also reported by Zaman et al.^22^

^h^ A(H5N1) virus infection in the case in Vietnam was serologically confirmed from a convalescent serum specimen with a neutralizing antibody titer of 160 by microneutralization assay to A(H5N1) virus, clade 2.3.2.1, and a hemagglutination inhibition antibody titer to the same virus of 160. A(H5N1) virus infection in the case in Pakistan was serologically confirmed from a convalescent serum specimen with a neutralizing antibody titer of 320 to A(H5N1) virus by microneutralization assay and a positive result on Western blot assay.

^i^ Reverse transcription–polymerase chain reaction cycle threshold value was not reported but virus was isolated by viral culture.

### MC Cases

Of 16 reported MC cases (14 adults, 2 children), 11 were identified by enhanced surveillance of persons exposed to A(H5N1) virus–infected poultry (8 in Bangladesh, 2 in Spain, and 1 in the UK). Enhanced surveillance programs included a study of individuals working at live bird markets in Bangladesh that included nasopharyngeal and oropharyngeal swab specimen collection from a sample of healthy workers and national avian influenza surveillance programs in Spain and the UK that included respiratory specimen collection from persons exposed to infected animals. The 8 reported cases in Bangladesh did not have serologic testing or additional respiratory specimen collection after testing positive.^17^ The 2 cases identified in Spain had initial positive nasopharyngeal specimens with high cycle threshold values and negative serology results (assay type and seropositivity criteria not reported); 1 case had a second negative respiratory specimen collected 16 days after the first positive specimen.^14^ Both cases in Spain were thought to reflect possible environmental contamination rather than true infection.^14^ The case identified in the UK was positive on 3 serial respiratory specimens over 7 days, all with cycle threshold values greater than 30; 2 of the positive specimens were taken after the case patient had started oseltamivir treatment.^20^ The remaining 5 MC cases (3 in Vietnam, 2 in Cambodia), including the only 2 cases in children, were identified by investigations of household contacts of index A(H5N1) case patients; 1 of these cases also had reported contact with dead chickens from a backyard flock as a possible source of infection.

## Discussion

Asymptomatic human infections with highly pathogenic avian influenza A(H5N1) virus, confirmed by molecular testing, have been infrequently reported, and most have lacked serologic confirmation. Although molecular detection alone, especially with high cycle threshold values by RT-PCR indicating low viral RNA levels, could represent environmental contamination or transient upper airway detection,^14^ the 2 case patients identified with MSC, combined with detailed epidemiologic investigations, provide evidence that asymptomatic A(H5N1) virus infections have occurred.

### Limitations

This review has some limitations, including that data for some reported asymptomatic infections are unpublished,^23^ and the numbers of asymptomatic exposed persons with respiratory and serum specimens collected for A(H5N1) virus testing were unavailable to estimate risk. Only 2 reports of enhanced A(H5N1) surveillance provided denominator data, with reported molecular detection of 1% (1 of 84) and 4% (8 of 192) among asymptomatic persons with animal exposures.^15,17^ In addition, methods for symptom ascertainment among reported asymptomatic cases ranged from ascertainment at a single time point (which could lead to misclassification if symptoms developed later) to varying intervals of follow-up after initial case detection.

## Conclusions

Prospective surveillance studies with serial respiratory and serum sampling and detailed symptom monitoring in persons with high-risk exposures to infected animals or close contact with humans with confirmed A(H5N1) virus infection could elucidate the true fractions of human A(H5N1) virus infections that are asymptomatic with and without seroconversion and the duration of viral shedding with asymptomatic infection. Careful attention to the timing of initiation of influenza antiviral postexposure prophylaxis and antiviral treatment would be needed when interpreting results. Such studies could provide information to guide risk assessment, infection control guidance, and population immunity projections but are resource intensive and may be unacceptable in some populations. Whether persons with asymptomatic A(H5N1) virus infections are a transmission risk to their close contacts is an additional critical knowledge gap. This review highlights the need for robust data collection from persons with possible asymptomatic A(H5N1) virus infection to inform future public health responses.

## Notes

Supplement 1. **eTable 1. **Search Strategy and Results for Initial Search to Include Publications Through January 31, 2025 **eTable 2. **Search Strategy and Results for Search Update to Include Publications From January 1, 2024 Through August 25, 2025

Supplement 2.
Data Sharing Statement

## References

[1] Cumulative number of confirmed human cases for avian influenza A(H5N1) reported to WHO, 2003-2025, 25 August 2025. World Health Organization. Accessed September 22, 2025. https://www.who.int/publications/m/item/cumulative-number-of-confirmed-human-cases-for-avian-influenza-a(h5n1)-reported-to-who--2003-2025--25-august-2025

[2] Chan PK. Outbreak of avian influenza A(H5N1) virus infection in Hong Kong in 1997. Clin Infect Dis. 2002;34(suppl 2):S58-S64. doi:10.1086/338820 11938498

[3] Peiris JS, Yu WC, Leung CW, . Re-emergence of fatal human influenza A subtype H5N1 disease. Lancet. 2004;363(9409):617-619. doi:10.1016/S0140-6736(04)15595-5 14987888 PMC7112424

[4] Dinh PN, Long HT, Tien NT, ; World Health Organization/Global Outbreak Alert and Response Network Avian Influenza Investigation Team in Vietnam. Risk factors for human infection with avian influenza A H5N1, Vietnam, 2004. Emerg Infect Dis. 2006;12(12):1841-1847. doi:10.3201/eid1212.060829 17326934 PMC3291373

[5] Zhou L, Liao Q, Dong L, . Risk factors for human illness with avian influenza A (H5N1) virus infection in China. J Infect Dis. 2009;199(12):1726-1734. doi:10.1086/599206 19416076 PMC2759027

[6] Lai S, Qin Y, Cowling BJ, . Global epidemiology of avian influenza A H5N1 virus infection in humans, 1997-2015: a systematic review of individual case data. Lancet Infect Dis. 2016;16(7):e108-e118. doi:10.1016/S1473-3099(16)00153-5 27211899 PMC4933299

[7] Van Kerkhove MD, Riley S, Lipsitch M, . Comment on “Seroevidence for H5N1 influenza infections in humans: meta-analysis”. Science. 2012;336(6088):1506. doi:10.1126/science.1221434 22723396

[8] Montgomery MP, Morris SE, Rolfes MA, . The role of asymptomatic infections in influenza transmission: what do we really know. Lancet Infect Dis. 2024;24(6):e394-e404. doi:10.1016/S1473-3099(23)00619-9 38128563 PMC11127787

[9] Tricco AC, Lillie E, Zarin W, et al. PRISMA extension for scoping reviews (PRISMA-ScR): checklist and explanation. Ann Intern Med. 2018,169(7):467-473. doi:10.7326/M18-0850 30178033

[10] WHO case definition for human infections with avian influenza A(H5) virus requiring notification under IHR (2005). World Health Organization. November 4, 2024. Accessed April 15, 2025. https://www.who.int/teams/global-influenza-programme/avian-influenza/case-definitions

[11] Powell TJ, Fox A, Peng Y, . Identification of H5N1-specific T-cell responses in a high-risk cohort in Vietnam indicates the existence of potential asymptomatic infections. J Infect Dis. 2012;205(1):20-27. doi:10.1093/infdis/jir689 22080094 PMC3242740

[12] Peters MDJ, Godfrey C, McInerney P, Munn Z, Tricco AC, Khalil H. Scoping reviews. In: Aromataris E, Lockwood C, Porritt K, Pilla B, Jordan Z, eds. *JBI Manual for Evidence Synthesis*. JBI; 2024. Accessed June 1, 2025. https://jbi-global-wiki.refined.site/space/MANUAL

[13] Alexakis L, Buczkowski H, Ducatez M, ; European Food Safety Authority, European Centre for Disease Prevention and Control, European Union Reference Laboratory for Avian Influenza. Avian influenza overview June-September 2024. EFSA J. 2024;22(10):e9057. 39434784 10.2903/j.efsa.2024.9057 PMC11492803

[14] Aznar E, Casas I, González Praetorius A, . Influenza A(H5N1) detection in two asymptomatic poultry farm workers in Spain, September to October 2022: suspected environmental contamination. Euro Surveill. 2023;28(8):2300107. doi:10.2807/1560-7917.ES.2023.28.8.2300107 36820643 PMC9951258

[15] Capelastegui F, Smith J, Kumbang J, . Pilot of asymptomatic swabbing of humans following exposures to confirmed avian influenza A(H5) in avian species in England, 2021/2022. Influenza Other Respir Viruses. 2023;17(8):e13187. doi:10.1111/irv.13187 37638093 PMC10447230

[16] Fusaro A, Gonzales JL, Kuiken T, ; European Food Safety Authority; European Centre for Disease Prevention and Control; European Union Reference Laboratory for Avian Influenza. Avian influenza overview December 2023-March 2024. EFSA J. 2024;22(3):e8754. 38550271 10.2903/j.efsa.2024.8754 PMC10977096

[17] Hassan MZ, Sturm-Ramirez K, Islam MS, . Interpretation of molecular detection of avian influenza A virus in respiratory specimens collected from live bird market workers in Dhaka, Bangladesh: infection or contamination? Int J Infect Dis. 2023;136:22-28. doi:10.1016/j.ijid.2023.08.020 37652093 PMC11849796

[18] Human cases of avian influenza A (H5N1) in North-West Frontier Province, Pakistan, October-November 2007. Wkly Epidemiol Rec. 2008;83(40):359-364. 18833663

[19] Le MQ, Horby P, Fox A, . Subclinical avian influenza A(H5N1) virus infection in human, Vietnam. Emerg Infect Dis. 2013;19(10):1674-1677. doi:10.3201/eid1910.130730 24047510 PMC3810763

[20] Oliver I, Roberts J, Brown CS, . A case of avian influenza A(H5N1) in England, January 2022. Euro Surveill. 2022;27(5):2200061. doi:10.2807/1560-7917.ES.2022.27.5.2200061 35115075 PMC8815099

[21] Olsen SJ, Ungchusak K, Sovann L, . Family clustering of avian influenza A (H5N1). Emerg Infect Dis. 2005;11(11):1799-1801. doi:10.3201/eid1111.050646 16422010 PMC3367331

[22] Zaman M, Ashraf S, Dreyer NA, Toovey S. Human infection with avian influenza virus, Pakistan, 2007. Emerg Infect Dis. 2011;17(6):1056-1059. doi:10.3201/eid/1706.091652 21749769 PMC3358180

[23] Investigation into the risk to human health of avian influenza (influenza A H5N1) in England: technical briefing 5. UK Health Security Agency. Updated July 14, 2023. Accessed August 11, 2025. https://www.gov.uk/government/publications/avian-influenza-influenza-a-h5n1-technical-briefings/investigation-into-the-risk-to-human-health-of-avian-influenza-influenza-a-h5n1-in-england-technical-briefing-5

