# Supplementary material for: Asymptomatic Human Infections With Avian Influenza A(H5N1) Virus Confirmed by Molecular and Serologic Testing: A Scoping Review
Source: JAMA Netw Open. 2025 Oct 29;8(10):e2540249. doi: 10.1001/jamanetworkopen.2025.40249 (PMC12573033; doi:10.1001/jamanetworkopen.2025.40249)
Supplement: Supplement 1. — eTable 1. Search Strategy and Results for Initial Search to Include Publications Through January 31, 2025 eTable 2. Search Strategy and Results for Search Update to Include Publications From January 1, 2024 Through August 25, 2025 [file jamanetwopen-e2540249-s001.pdf]

Supplementary Online Content

Dawood FS, Garg S, Patel P, Uyeki TM. Asymptomatic human infections with influenza A(H5N1) virus confirmed by molecular and serologic testing: a scoping review. *JAMA Netw Open.* 2025;8(10):e2540249. doi:10.1001/jamanetworkopen.2025.40249

eTable 1. Search Strategy and Results for Initial Search to Include Publications Through January 31, 2025

eTable 2. Search Strategy and Results for Search Update to Include Publications From January 1, 2024 Through August 25, 2025

This supplementary material has been provided by the authors to give readers additional information about their work.

eTable 1. Search Strategy and Results for Initial Search to Include Publications Through January 31, 2025

Database	Strategy	Run Date	Records
Medline (Ovid) 1946-	(h5n1 OR "h5 n1" OR ah5n1).mp. OR exp Influenza A Virus, H5N1 Subtype/<br><br>AND<br><br>(asymptom* OR pre symptom* OR presymptom* OR symptomless* OR symptom less* OR nonsymptom* OR non symptom* OR subclinical* OR "sub clinical" OR first case* OR first report* OR "person to person" OR "human to human" OR carrier state* OR supatent* OR submicroscopic*).mp. OR ((silent* OR carrier*) adj2 (infect*)).mp. OR ((spread* OR superspreader* OR inapparent* OR unapparent* OR silent*) adj4 (carrier*)).mp. OR ((human* OR people* OR person* OR communit*) adj4 (transmi* OR case* OR infect* OR outbreak*)).ti. OR exp Asymptomatic Infections/ OR exp Carrier State/<br><br>Exclude animals	2025-01-31	672 articles<br><br>Duplicates identified by EndNote<br><br>= 668 articles
Medline (PubMed) 1946-	TI,AB(h5n1 OR "h5 n1" OR ah5n1) OR "Influenza A Virus, H5N1 Subtype"[Mesh]<br><br>AND<br><br>TI,AB(asymptomatic OR presymptomatic OR presymptomatics OR symptomless OR presymptom OR presymptoms OR nonsymptomatic OR subclinical OR "pre symptom" OR "pre symptoms" OR "pre symptomatic" OR "symptom less" OR "non symptomatic" OR "sub clinical" OR "first case" OR "first report" OR "first cases" OR "first reports" OR "person to person" OR "human to human" OR "carrier state" OR "carrier states" OR "infection carrier" OR "infection carriers" OR "spreader carrier" OR "spreader carriers" OR "superspreader carrier" OR "superspreader carriers" OR "inapparent carrier" OR "unapparent carrier" OR "inapparent carriers" OR "unapparent carriers" OR "silent carrier" OR "silent carriers" OR supatent OR submicroscopic OR "silent infection" OR "silent infections") OR TI((human OR humans OR people OR person OR persons OR community OR communities) AND (transmission OR case OR cases OR infect OR infection OR		655 articles<br><br>Duplicates identified by EndNote<br><br>=62 articles

© 2025 Dawood FS et al. *JAMA Network Open.*

	infected OR infecting OR outbreak OR outbreaks)) OR "Asymptomatic Infections"[Mesh] OR "Carrier State"[Mesh]<br><br>Exclude animals		
Embase (Ovid)<br>1947-	(h5n1 OR "h5 n1" OR ah5n1).mp. OR exp "avian influenza (h5n1)"/<br><br>AND<br><br>(asymptom* OR pre symptom* OR presymptom* OR symptomless* OR symptom less* OR nonsymptom* OR non symptom* OR subclinical* OR "sub clinical" OR first case* OR first report* OR "person to person" OR "human to human" OR carrier state* OR supatent* OR submicroscopic*).mp. OR ((silent* OR carrier*) adj2 (infect*)).mp. OR ((spread* OR superspreader* OR inapparent* OR unapparent* OR silent*) adj4 (carrier*)).mp. OR ((human* OR people* OR person* OR communit*) adj4 (transmi* OR case* OR infect* OR outbreak*)).ti. OR exp asymptomatic carrier/ or asymptomatic infection/ or asymptomatic disease/ or exp asymptomatic transmission/ OR exp subpatent infection/<br><br>Exclude animals		792 articles<br><br>Duplicates identified by EndNote<br><br>=289 articles
Global Health (Ovid)<br>1910-	(h5n1 OR "h5 n1" OR ah5n1).mp.<br><br>AND<br><br>(asymptom* OR pre symptom* OR presymptom* OR symptomless* OR symptom less* OR nonsymptom* OR non symptom* OR subclinical* OR "sub clinical" OR first case* OR first report* OR "person to person" OR "human to human" OR carrier state* OR supatent* OR submicroscopic*).mp. OR ((silent* OR carrier*) adj2 (infect*)).mp. OR ((spread* OR superspreader* OR inapparent* OR unapparent* OR silent*) adj4 (carrier*)).mp. OR ((human* OR people* OR person* OR communit*) adj4 (transmi* OR case* OR infect* OR outbreak*)).ti. OR asymptomatic infections/ OR exp carrier state/		531 articles<br><br>Duplicates identified by EndNote<br><br>=190 articles
Cochrane Library	(h5n1 OR "h5 n1" OR ah5n1):ti,ab<br><br>AND		9 articles

© 2025 Dawood FS et al. *JAMA Network Open.*

	(asymptom* OR presymptom* OR symptomless* OR nonsymptom* OR subclinical* OR "pre symptom" OR "pre symptoms" OR "pre symptomatic" OR "symptom less" OR "non symptomatic" OR "sub clinical" OR "first case" OR "first report" OR "first cases" OR "first reports" OR "person to person" OR "human to human" OR "carrier state" OR "carrier states" OR supatent OR submicroscopic):ti,ab OR ((silent* OR carrier*) NEAR/2 (infect*)):ti,ab OR ((spread* OR superspreader* OR inapparent* OR unapparent* OR silent*) NEAR/4 (carrier*)):ti,ab OR ((human* OR people* OR person* OR communit*) NEAR/4 (transmi* OR case* OR infect* OR outbreak*)):ti		Duplicates identified by EndNote<br><br>=5 articles
Scopus 1960-	TITLE-ABS-KEY(h5n1 OR "h5 n1" OR ah5n1)<br><br>AND<br><br>TITLE-ABS-KEY(asymptom* OR presymptom* OR symptomless* OR nonsymptom* OR subclinical* OR "pre symptom" OR "pre symptoms" OR "pre symptomatic" OR "symptom less" OR "non symptomatic" OR "sub clinical" OR "first case" OR "first report" OR "first cases" OR "first reports" OR "person to person" OR "human to human" OR "carrier state" OR "carrier states" OR supatent OR submicroscopic) OR TITLE-ABS-KEY((silent* OR carrier*) W/2 (infect*)) OR TITLE-ABS-KEY((spread* OR superspreader* OR inapparent* OR unapparent* OR silent*) W/4 (carrier*)) OR TITLE((human* OR people* OR person* OR communit*) W/4 (transmi* OR case* OR infect* OR outbreak*))		995 articles<br><br>Duplicates identified by EndNote<br><br>=224 articles
PubMed Central	TI,AB(h5n1 OR "h5 n1" OR ah5n1) OR "Influenza A Virus, H5N1 Subtype"[Mesh]<br><br>AND<br><br>TI,AB(asymptomatic OR presymptomatic OR presymptomatics OR symptomless OR presymptom OR presymptoms OR nonsymptomatic OR subclinical OR "pre symptom" OR "pre symptoms" OR "pre symptomatic" OR "symptom less" OR "non symptomatic" OR "sub clinical" OR "first case" OR "first report" OR "first cases" OR "first reports" OR "person to person" OR "human to human" OR "carrier state" OR "carrier states" OR		338 articles<br><br>Duplicates identified by EndNote<br><br>=17 articles

© 2025 Dawood FS et al. *JAMA Network Open.*

	"infection carrier" OR "infection carriers" OR "spreader carrier" OR "spreader carriers" OR "superspreader carrier" OR "superspreader carriers" OR "inapparent carrier" OR "unapparent carrier" OR "inapparent carriers" OR "unapparent carriers" OR "silent carrier" OR "silent carriers" OR supatent OR submicroscopic OR "silent infection" OR "silent infections") OR TI((human OR humans OR people OR person OR persons OR community OR communities) AND (transmission OR case OR cases OR infect OR infection OR infected OR infecting OR outbreak OR outbreaks)) OR "Asymptomatic Infections"[Mesh] OR "Carrier State"[Mesh]<br><br>Exclude animals		
Global Index Medicus	TI,AB(h5n1 OR "h5 n1" OR ah5n1)<br><br>AND<br><br>TI,AB(asymptom* OR presymptom* OR symptomless* OR nonsymptom* OR subclinical* OR "pre symptom" OR "pre symptoms" OR "pre symptomatic" OR "symptom less" OR "non symptomatic" OR "sub clinical" OR "first case" OR "first report" OR "first cases" OR "first reports" OR "person to person" OR "human to human" OR "carrier state" OR "carrier states" OR "infection carrier" OR "infection carriers" OR "spreader carrier" OR "spreader carriers" OR "superspreader carrier" OR "superspreader carriers" OR "inapparent carrier" OR "unapparent carrier" OR "inapparent carriers" OR "unapparent carriers" OR "silent carrier" OR "silent carriers" OR supatent OR submicroscopic OR "silent infection" OR "silent infections") OR TI((human OR humans OR people OR person OR persons OR community OR communities) AND (transmission OR case OR cases OR infect OR infection OR infected OR infecting OR outbreak OR outbreaks))<br><br>Exclude animals		48 articles<br><br>Duplicates identified by EndNote<br><br>=28 articles
Virtual Health Library	TI,AB(h5n1 OR "h5 n1" OR ah5n1)<br><br>AND<br><br>TI,AB(asymptom* OR presymptom* OR symptomless* OR nonsymptom* OR subclinical* OR "pre symptom" OR "pre symptoms" OR "pre symptomatic" OR "symptom less" OR "non		347 articles<br><br>Duplicates identified by EndNote<br><br>=28 articles

	symptomatic" OR "sub clinical" OR "first case" OR "first report" OR "first cases" OR "first reports" OR "person to person" OR "human to human" OR "carrier state" OR "carrier states" OR "infection carrier" OR "infection carriers" OR "spreader carrier" OR "spreader carriers" OR "superspreader carrier" OR "superspreader carriers" OR "inapparent carrier" OR "unapparent carrier" OR "inapparent carriers" OR "unapparent carriers" OR "silent carrier" OR "silent carriers" OR supatent OR submicroscopic OR "silent infection" OR "silent infections") OR TI((human OR humans OR people OR person OR persons OR community OR communities) AND (transmission OR case OR cases OR infect OR infection OR infected OR infecting OR outbreak OR outbreaks))<br><br>Exclude animals		
EuropePMC: Preprints	TI,AB(h5n1 OR "h5 n1" OR ah5n1)<br><br>AND<br><br>TI,AB(asymptom* OR presymptom* OR symptomless* OR nonsymptom* OR subclinical* OR "pre symptom" OR "pre symptoms" OR "pre symptomatic" OR "symptom less" OR "non symptomatic" OR "sub clinical" OR "first case" OR "first report" OR "first cases" OR "first reports" OR "person to person" OR "human to human" OR "carrier state" OR "carrier states" OR "infection carrier" OR "infection carriers" OR "spreader carrier" OR "spreader carriers" OR "superspreader carrier" OR "superspreader carriers" OR "inapparent carrier" OR "unapparent carrier" OR "inapparent carriers" OR "unapparent carriers" OR "silent carrier" OR "silent carriers" OR supatent OR submicroscopic OR "silent infection" OR "silent infections") OR TI((human OR humans OR people OR person OR persons OR community OR communities) AND (transmission OR case OR cases OR infect OR infection OR infected OR infecting OR outbreak OR outbreaks))<br><br>Limits: preprints only		36 articles<br><br>Duplicates identified by EndNote<br><br>=28 articles
Weekly Epidemiological Report (WHO)			Not available at time of search

© 2025 Dawood FS et al. *JAMA Network Open.*

eTable 2. Search Strategy and Results for Search Update to Include Publications From January 1, 2024 Through August 25, 2025

Database	Strategy	Run Date	Records
Medline (Ovid) 1946-	(h5n1 OR "h5 n1" OR ah5n1).mp. OR exp Influenza A Virus, H5N1 Subtype/<br><br>AND<br><br>(asymptom* OR pre symptom* OR presymptom* OR symptomless* OR symptom less* OR nonsymptom* OR non symptom* OR subclinical* OR "sub clinical" OR first case* OR first report* OR "person to person" OR "human to human" OR carrier state* OR supatent* OR submicroscopic*).mp. OR ((silent* OR carrier*) adj2 (infect*)).mp. OR ((spread* OR superspreader* OR inapparent* OR unapparent* OR silent*) adj4 (carrier*)).mp. OR ((human* OR people* OR person* OR communit*) adj4 (transmi* OR case* OR infect* OR outbreak*)).ti. OR exp Asymptomatic Infections/ OR exp Carrier State/<br><br>Exclude animals	2025-08-25	105 articles<br><br>- duplicates<br><br>= 104 articles
Medline (PubMed) 1946-	TI,AB(h5n1 OR "h5 n1" OR ah5n1) OR "Influenza A Virus, H5N1 Subtype"[Mesh]<br><br>AND<br><br>TI,AB(asymptomatic OR presymptomatic OR presymptomatics OR symptomless OR presymptom OR presymptoms OR nonsymptomatic OR subclinical OR "pre symptom" OR "pre symptoms" OR "pre symptomatic" OR "symptom less" OR "non symptomatic" OR "sub clinical" OR "first case" OR "first report" OR "first cases" OR "first reports" OR "person to person" OR "human to human" OR "carrier state" OR "carrier states" OR "infection carrier" OR "infection carriers" OR "spreader carrier" OR "spreader carriers" OR "superspreader carrier" OR "superspreader carriers" OR "inapparent carrier" OR "unapparent carrier" OR "inapparent carriers" OR "unapparent carriers" OR "silent carrier" OR "silent carriers" OR supatent OR submicroscopic OR "silent infection" OR "silent infections") OR TI((human OR humans OR people OR person OR persons OR community OR communities) AND (transmission OR case OR cases OR infect OR infection OR infected OR infecting OR outbreak OR		98 articles<br><br>- duplicates<br><br>=8 articles

© 2025 Dawood FS et al. *JAMA Network Open.*

	outbreaks)) OR "Asymptomatic Infections"[Mesh] OR "Carrier State"[Mesh]<br><br>Exclude animals		
Embase (Ovid) 1947-	(h5n1 OR "h5 n1" OR ah5n1).mp. OR exp "avian influenza (h5n1)"/<br><br>AND<br><br>(asymptom* OR pre symptom* OR presymptom* OR symptomless* OR symptom less* OR nonsymptom* OR non symptom* OR subclinical* OR "sub clinical" OR first case* OR first report* OR "person to person" OR "human to human" OR carrier state* OR supatent* OR submicroscopic*).mp. OR ((silent* OR carrier*) adj2 (infect*)).mp. OR ((spread* OR superspreader* OR inapparent* OR unapparent* OR silent*) adj4 (carrier*)).mp. OR ((human* OR people* OR person* OR communit*) adj4 (transmi* OR case* OR infect* OR outbreak*)).ti. OR exp asymptomatic carrier/ or asymptomatic infection/ or asymptomatic disease/ or exp asymptomatic transmission/ OR exp subpatent infection/<br><br>Exclude animals		122 articles<br><br>- duplicates<br><br>=36 articles
Global Health (Ovid) 1910-	(h5n1 OR "h5 n1" OR ah5n1).mp.<br><br>AND<br><br>(asymptom* OR pre symptom* OR presymptom* OR symptomless* OR symptom less* OR nonsymptom* OR non symptom* OR subclinical* OR "sub clinical" OR first case* OR first report* OR "person to person" OR "human to human" OR carrier state* OR supatent* OR submicroscopic*).mp. OR ((silent* OR carrier*) adj2 (infect*)).mp. OR ((spread* OR superspreader* OR inapparent* OR unapparent* OR silent*) adj4 (carrier*)).mp. OR ((human* OR people* OR person* OR communit*) adj4 (transmi* OR case* OR infect* OR outbreak*)).ti. OR asymptomatic infections/ OR exp carrier state/		43 articles<br><br>- duplicates<br><br>=4 articles
Cochrane Library	(h5n1 OR "h5 n1" OR ah5n1):ti,ab<br><br>AND<br><br>(asymptom* OR presymptom* OR symptomless* OR nonsymptom* OR subclinical* OR "pre symptom" OR "pre symptoms" OR "pre symptomatic" OR "symptom less" OR "non symptomatic" OR "sub clinical" OR "first case" OR "first report" OR "first cases" OR "first reports" OR "person to person" OR "human to human" OR		0 articles<br><br>- duplicates<br><br>=0 articles

© 2025 Dawood FS et al. *JAMA Network Open.*

	"carrier state" OR "carrier states" OR supatent OR submicroscopic):ti,ab OR ((silent* OR carrier*) NEAR/2 (infect*)):ti,ab OR ((spread* OR superspreader* OR inapparent* OR unapparent* OR silent*) NEAR/4 (carrier*)):ti,ab OR ((human* OR people* OR person* OR communit*) NEAR/4 (transmi* OR case* OR infect* OR outbreak*)):ti		
Scopus 1960-	TITLE-ABS-KEY(h5n1 OR "h5 n1" OR ah5n1)<br><br>AND<br><br>TITLE-ABS-KEY(asymptom* OR presymptom* OR symptomless* OR nonsymptom* OR subclinical* OR "pre symptom" OR "pre symptoms" OR "pre symptomatic" OR "symptom less" OR "non symptomatic" OR "sub clinical" OR "first case" OR "first report" OR "first cases" OR "first reports" OR "person to person" OR "human to human" OR "carrier state" OR "carrier states" OR supatent OR submicroscopic) OR TITLE-ABS-KEY((silent* OR carrier*) W/2 (infect*)) OR TITLE-ABS-KEY((spread* OR superspreader* OR inapparent* OR unapparent* OR silent*) W/4 (carrier*)) OR TITLE((human* OR people* OR person* OR communit*) W/4 (transmi* OR case* OR infect* OR outbreak*))		106 articles<br><br>- duplicates<br><br>=4 articles
PubMed Central	TI,AB(h5n1 OR "h5 n1" OR ah5n1) OR "Influenza A Virus, H5N1 Subtype"[Mesh]<br><br>AND<br><br>TI,AB(asymptomatic OR presymptomatic OR presymptomatics OR symptomless OR presymptom OR presymptoms OR nonsymptomatic OR subclinical OR "pre symptom" OR "pre symptoms" OR "pre symptomatic" OR "symptom less" OR "non symptomatic" OR "sub clinical" OR "first case" OR "first report" OR "first cases" OR "first reports" OR "person to person" OR "human to human" OR "carrier state" OR "carrier states" OR "infection carrier" OR "infection carriers" OR "spreader carrier" OR "spreader carriers" OR "superspreader carrier" OR "superspreader carriers" OR "inapparent carrier" OR "unapparent carrier" OR "inapparent carriers" OR "unapparent carriers" OR "silent carrier" OR "silent carriers" OR supatent OR submicroscopic OR "silent infection" OR "silent infections") OR TI((human OR humans OR people OR person OR persons OR community OR communities) AND (transmission OR case OR cases OR infect OR infection OR infected OR infecting OR outbreak OR		88 articles<br><br>- duplicates<br><br>=2 articles

© 2025 Dawood FS et al. *JAMA Network Open.*

	outbreaks)) OR "Asymptomatic Infections"[Mesh] OR "Carrier State"[Mesh]<br><br>Exclude animals		
Global Index Medicus	TI,AB("h5n1" OR "h5 n1" OR "ah5n1"<br><br>AND<br><br>TI,AB(asymptom* OR presymptom* OR symptomless* OR nonsymptom* OR subclinical* OR "pre symptom" OR "pre symptoms" OR "pre symptomatic" OR "symptom less" OR "non symptomatic" OR "sub clinical" OR "first case" OR "first report" OR "first cases" OR "first reports" OR "person to person" OR "human to human" OR "carrier state" OR "carrier states" OR "infection carrier" OR "infection carriers" OR "spreader carrier" OR "spreader carriers" OR "superspreader carrier" OR "superspreader carriers" OR "inapparent carrier" OR "unapparent carrier" OR "inapparent carriers" OR "unapparent carriers" OR "silent carrier" OR "silent carriers" OR supatent OR submicroscopic OR "silent infection" OR "silent infections") OR TI((human OR humans OR people OR person OR persons OR community OR communities) AND (transmission OR case OR cases OR infect OR infection OR infected OR infecting OR outbreak OR outbreaks))<br><br>Exclude animals		0 articles<br><br>- duplicates<br><br>=0 articles
Virtual Health Library	TI,AB("h5n1" OR "h5 n1" OR "ah5n1")<br><br>AND<br><br>TI,AB(asymptom* OR presymptom* OR symptomless* OR nonsymptom* OR subclinical* OR "pre symptom" OR "pre symptoms" OR "pre symptomatic" OR "symptom less" OR "non symptomatic" OR "sub clinical" OR "first case" OR "first report" OR "first cases" OR "first reports" OR "person to person" OR "human to human" OR "carrier state" OR "carrier states" OR "infection carrier" OR "infection carriers" OR "spreader carrier" OR "spreader carriers" OR "superspreader carrier" OR "superspreader carriers" OR "inapparent carrier" OR "unapparent carrier" OR "inapparent carriers" OR "unapparent carriers" OR "silent carrier" OR "silent carriers" OR supatent OR submicroscopic OR "silent infection" OR "silent infections") OR TI((human OR humans OR people OR person OR persons OR community OR communities) AND (transmission OR case OR cases OR infect OR infection OR infected OR infecting OR outbreak OR outbreaks))		46 articles<br><br>- duplicates<br><br>=24 articles

© 2025 Dawood FS et al. *JAMA Network Open.*

	Exclude animals		
EuropePMC: Preprints	TI,AB(h5n1 OR "h5 n1" OR ah5n1)<br><br>AND<br><br>TI,AB(asymptom* OR presymptom* OR symptomless* OR nonsymptom* OR subclinical* OR "pre symptom" OR "pre symptoms" OR "pre symptomatic" OR "symptom less" OR "non symptomatic" OR "sub clinical" OR "first case" OR "first report" OR "first cases" OR "first reports" OR "person to person" OR "human to human" OR "carrier state" OR "carrier states" OR "infection carrier" OR "infection carriers" OR "spreader carrier" OR "spreader carriers" OR "superspreader carrier" OR "superspreader carriers" OR "inapparent carrier" OR "unapparent carrier" OR "inapparent carriers" OR "unapparent carriers" OR "silent carrier" OR "silent carriers" OR supatent OR submicroscopic OR "silent infection" OR "silent infections") OR TI((human OR humans OR people OR person OR persons OR community OR communities) AND (transmission OR case OR cases OR infect OR infection OR infected OR infecting OR outbreak OR outbreaks))<br><br>Limits: preprints only		36 articles<br><br>- duplicates<br><br>=0 articles

© 2025 Dawood FS et al. *JAMA Network Open.*
