# Supplementary material for: Asymptomatic Human Infections With Avian Influenza A(H5N1) Virus Confirmed by Molecular and Serologic Testing: A Scoping Review
Source: JAMA Netw Open. 2025 Oct 29;8(10):e2540249. doi: 10.1001/jamanetworkopen.2025.40249 (PMC12573033; doi:10.1001/jamanetworkopen.2025.40249)
Supplement: Supplement 2. — Data Sharing Statement [file jamanetwopen-e2540249-s002.pdf]

Data Sharing Statement

Dawood. Asymptomatic Human Infections With Influenza A(H5N1) Virus Confirmed by Molecular and Serologic Testing. *JAMA Netw Open*. Published October 29, 2025. doi:10.1001/jamanetworkopen.2025.40249

Data
Data available: No

Additional Information
Explanation for why data not available: Not applicable.
